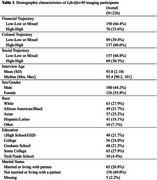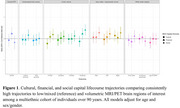# Association of life course socioeconomic status with late‐life neuroimaging biomarkers among the oldest‐old

**DOI:** 10.1002/alz70856_106080

**Published:** 2026-01-09

**Authors:** Hilary L. Colbeth, Rachel L. Peterson, Kristen M. George, Alexander Ivan B. Posis, Rifat B. Alam, Paola Gilsanz, María M. M. Corrada, Rachel A. Whitmer

**Affiliations:** ^1^ University of California, Davis, Davis, CA, USA; ^2^ University of Montana, Missoula, MT, USA; ^3^ Kaiser Permanente Northern California Division of Research, Pleasanton, CA, USA; ^4^ University of California, Irvine, Irvine, CA, USA

## Abstract

**Background:**

Socioeconomic status (SES) is a multidimensional construct that includes measures of financial status, education, and social prestige. Few studies have examined dimensions of SES across the lifecourse in relation to neurodegeneration into the oldest ages (90+ years).

**Methods:**

*LifeAfter90* is an ethno‐racially diverse cohort study of adults ages 90+ in northern California. We evaluated the associations of three SES domains (cultural, financial, and social capital) with volumetric MRI and PET brain regions of interest (ROI). We defined high childhood financial capital as at least one parent working full‐time and high adulthood financial capital as reporting little worry about expenses; high childhood cultural capital as one parent completing some college or more and high adulthood cultural capital as the participant completing some college or more; high childhood social capital as rating their family ≥ 5 on the MacArthur Scale of Subjective Social Status (range 1‐10) during childhood, and high adulthood social capital as reporting a close relationship. For each domain, we operationalized lifecourse SES domain‐specific capital as a binary variable comparing consistently high (i.e. high capital in childhood and adulthood) to a reference of consistently low/mixed capital. We used inverse probability of selection weighted linear regression models to estimate associations comparing high to low/mixed trajectories for each brain ROI, adjusting for age and sex/gender.

**Results:**

Among 226 participants (mean age 93±2.1), 56% were female, and 74% had at least some college education. For cultural capital, there were no significant differences between those with a consistently high trajectory and low/mixed trajectories (Figure 1). For financial capital, we observed higher amyloid (0.2096, 95% CI: ‐0.1002, 0.5193) and temporal cortex volumes (0.18, 95% CI: ‐0.087, 0.4469) among those with a consistently high financial trajectory vs low/mixed trajectories. For social capital, a consistently high social trajectory was significantly associated with larger temporal cortex volume (0.4367, 95% CI: 0.172, 0.7014), total gray matter (0.3023, 95% CI: 0.0204, 0.5841), and right hippocampal volumes (0.2893, 95% CI: 0.0089, 0.5698) vs low/mixed trajectories.

**Conclusions:**

Our findings indicate that lifecourse experiences into very old age of high social capital may impact ROIs specific to processing, emotion, and memory.